# The Spatial Architecture of *Bacillus subtilis* Biofilms Deciphered Using a Surface-Associated Model and *In Situ* Imaging

**DOI:** 10.1371/journal.pone.0016177

**Published:** 2011-01-18

**Authors:** Arnaud Bridier, Dominique Le Coq, Florence Dubois-Brissonnet, Vincent Thomas, Stéphane Aymerich, Romain Briandet

**Affiliations:** 1 INRA, UMR 1319 MICALIS, Jouy-en-Josas, France; 2 AgroParisTech, UMR 1319 MICALIS, Jouy-en-Josas, France; 3 Steris, Fontenay aux Roses, France; Loyola University Medical Center, United States of America

## Abstract

The formation of multicellular communities known as biofilms is the part of bacterial life cycle in which bacteria display cooperative behaviour and differentiated phenotypes leading to specific functions. *Bacillus subtilis* is a Gram-positive bacterium that has served for a decade as a model to study the molecular pathways that control biofilm formation. Most of the data on *B. subtilis* biofilms have come from studies on the formation of pellicles at the air-liquid interface, or on the complex macrocolonies that develop on semi-solid nutritive agar. Here, using confocal laser scanning microcopy, we show that *B. subtilis* strains of different origins are capable of forming biofilms on immersed surfaces with dramatically protruding “beanstalk-like” structures with certain strains. Indeed, these structures can reach a height of more than 300 µm with one undomesticated strain from a medical environment. Using 14 GFP-labeled mutants previously described as affecting pellicle or complex colony formation, we have identified four genes whose inactivation significantly impeded immersed biofilm development, and one mutation triggering hyperbiofilm formation. We also identified mutations causing the three-dimensional architecture of the biofilm to be altered. Taken together, our results reveal that *B. subtilis* is able to form specific biofilm features on immersed surfaces, and that the development of these multicellular surface-associated communities involves regulation pathways that are common to those governing the formation of pellicle and/or complex colonies, and also some specific mechanisms. Finally, we propose the submerged surface-associated biofilm as another relevant model for the study of *B. subtilis* multicellular communities.

## Introduction

Bacteria can grow within multicellular, matrix-enclosed communities known as biofilms [1], [2], [3]. It is now largely accepted that biofilms constitute the predominant microbial lifestyle in natural and engineered ecosystems, enabling bacteria to develop coordinated architectural and survival strategies [4]. The involvement of biofilms in a large number of ecological and biotechnological processes [5], as well as in human infections [6], [7], [8], highlights the importance of gaining a clearer understanding of the formation, development and maintenance of these biological structures, so that their control can be improved.

*Bacillus subtilis* is a Gram-positive, motile, spore-forming bacterium which has served as a model for the molecular study of biofilm formation during the past ten years [9]. Complex mechanisms involved in biofilm formation and development have been elucidated using this bacterium. During biofilm development, motile single cells differentiate into aligned bundles of sessile attached cells [10], [11]. This transition is accompanied by the production of an exopolymeric matrix resulting from the expression of two operons: (i) the 15-gene *epsA-O* operon which encodes the proteins required for polysaccharide synthesis and acts as an inhibitor of motility (EpsE) [12], and (ii) the *yqxM-sipW-tasA* operon which encodes a major protein component of the matrix (TasA) and its transport machinery [13], [14]. Romero *et al.* [15] recently reported that TasA forms amyloid fibers that bind cells together and are essential in the formation of robust cohesive biofilms. Previous studies had shown that four pairs of global regulators: Spo0A/AbrB, SinI/SinR, SlrR/SlrA and DegS/DegU, play a key role in the formation and development of complex multicellular communities through the direct or indirect control of both of these operons and of motility-involved genes [14], [16], [17], [18], [19], [20], [21], [22]. A phosphorylated form of the major early sporulation transcription factor Spo0A represses AbrB which negatively regulates biofilm formation [17], [18]. The intermediate level of Spo0A-P also stimulates the transcription of SinI [23], [24], which binds to and inactivates SinR, a major regulator of biofilm formation that directly represses the *epsO-A* and *yqxM-sipW-tasA* operons [14], [19]. In addition to SinI, two other proteins, YlbF and YmcA, have been identified as antagonizing SinR activity when conditions are favorable for biofilm formation [19]. Recent studies also reported that SinR activity is negatively controlled by SlrR, the transcription of which is in turn repressed by SinR as a downstream consequence of Spo0A phosphorylation. SinR is inhibited successively by SinI and SlrR, resulting in a de-repression of SinR direct targets (*slrR*, *eps*, and *yqxM*) and a repression of SlrR targets (*lytABC*, *lytF* – thus triggering chaining and also *hag* and possibly other σD-dependent motility genes) through formation of the SinR-SlrR complex [16], [25], [26], [20]. The transcription factor DegU also coordinates the multicellular behavior of *B. subtilis* by regulating motility, poly-γ-glutamic acid and protease production and the expression of other swarming- and biofilm-involved genes *via* a gradient in its phosphorylation level [21], [22], [27], [28], [29].

Interestingly, the vast majority of these genetic pathways, and other data on *B. subtilis* biofilms, were identified by studying the development of pellicle at the air-liquid interface or of complex macrocolonies on agar, but only very few studies have focused on surface-associated immersed biofilm models [17], [18], [28], [30]. Because some steps of biofilm formation (such as adhesion to the support) are specific to immersed surface-associated models, biofilm development under these conditions may involve regulation pathways than differ from those identified in pellicle and macrocolony models, leading to particular architectural features which govern specific functions.

In this context, we first of all characterized the structure of biofilms formed by different strains of *B. subtilis* on immersed surfaces using a microplate-based model combined with confocal laser scanning microscopy (CLSM). Then, using GFP-labeled strains, we studied the effects of mutations known to affect pellicle and complex colonies in our surface-associated biofilm model.

## Materials and Methods

### Strains and culture conditions

The *B. subtilis* strains used during this study are described in Table 1. The different strains were chosen in order to represent the diversity of *B.subtilis* strains and were thus selected from a variety of origins (collections and non-domesticated). The ATCC strains belonged to different subspecies: ATCC 6633 *B.subtilis* subsp spizizenii, ATCC 9372 *B.subtilis* subsp niger, ATCC 6051 *B.subtilis* subsp subtilis. Non-domesticated strains (ND_food_ and ND_medical_) had recently been isolated from foods (dessert cream) and medical environments (endoscope washer-disinfector) and typed as *B. subtilis* using biochemical identification methods and 16S rDNA sequencing. The GFP-carrying reference strain, GM2812, was obtained by transforming strain BSB168 (a *trp+* derivative of the reference strain 168 Marburg [31]) for spectinomycin resistance with the pDR146 plasmid (a kind gift from D. Rudner, Harvard Medical School). This placed in the *amyE* locus the *gfp (mut2)* gene controlled by the IPTG-regulated *P_hyperspank_* promoter. GM2812 derivatives mutated in various genes were then obtained by transformation with chromosomal DNA extracted from strains carrying the corresponding different alleles of interest marked with a suitable antibiotic resistance cassette. The extraction of chromosomal DNA, and the transformation of *B. subtilis*, were performed according to standard procedures, and the transformants were selected on LB plates supplemented with the relevant antibiotic at the following concentrations: spectinomycin, 100 µg ml^−1^; chloramphenicol, 4 µg ml^−1^; erythromycin, 0.5 µg ml^−1^; neomycin and kanamycin, 8µg ml^−1^. Before each experiment, the cells were subcultured in Tryptone Soya Broth (TSB, BioMérieux, France), supplemented with antibiotics when necessary.

**Table 1:** *Bacillus subtilis* strains used in this study.

Strain	Relevant genotype or description^a^	Origin or construction^b^
168	*trpC2*	Bacillus Genetic Stock Center
ATCC 6633		American Type Culture Collection
ATCC 9372		American Type Culture Collection
ATCC 6051		American Type Culture Collection
PG01		CTSCCV
ND_medical_		Non-domesticated, isolated from endoscope washer-disinfectors [35]
ND_food_		Non-domesticated, isolated from dairy product (ISHA)
BSB168	*trp+* derivative of 168	[31]
GM2812	*amyE*::P_hyp_-*gfp (spec)*	pDR146 (D. Rudner)→BSB168
GM2815	*amyE*::P_hyp_-*gfp (spec)*, *ΔdegQ*::*cat*	BG4138 [51]→GM2812
GM2817	*amyE*::P_hyp_-*gfp (spec)*, *degU*::*neo*	GM719 [52]→GM2812
GM2821	*amyE*::P_hyp_-*gfp (spec)*, *degU32*-*kan*	QB4371 [53]→GM2812
GM2828	*amyE*::P_hyp_-*gfp (spec)*, *yvfV*::*pMUTIN2 (ery)*	BSFA1123→GM2812
GM2830	*amyE*::P_hyp_-*gfp (spec)*, *yvfW*::*pMUTIN2 (ery)*	BSFA1124→GM2812
GM2832	*amyE*::P_hyp_-*gfp (spec)*, *gtaC*::*pBS640 (cat)*	L16601gtaC [54]→GM2812
GM2812ylbF1	*amyE*::P_hyp_-*gfp (spec)*, *ylbF*::*pMUTIN4 (ery)*	BSFA3233 [40]→GM2812
GM2812ymcA1	*amyE*::P_hyp_-*gfp (spec)*, *ymcA*::*pMUTIN4 (ery)*	BSFA2603 [40]→GM2812
GM2850	*amyE*::P_hyp_-*gfp (spec)*, *yxaB*::*pKN3 (ery)*	MM1701 [44]→GM2812
GM2851	*amyE*::P_hyp_-*gfp (spec)*, *ΔabrB*::*cat*	MM1717 [44]→GM2812
GM2853	*amyE*::P_hyp_-*gfp (spec)*, *ΔabrB*::*cat*, *yxaB*::*pKN3 (ery)*	MM1707 [44] àGM2812
GM2855	*amyE*::P_hyp_-*gfp (spec)*, *sigX*::*pMUTIN2 (ery)*	BSFA94→GM2812
GM2857	*amyE*::P_hyp_-*gfp (spec)*, *yqxM*::*pMUTIN3 (ery)*	BSFA4767 [40]→GM2812
GM2888	*amyE*::P_hyp_-*gfp (spec)*, *Δhag*::*cat*	OMG954 [55]→GM2812

a *spec*, *cat*, *neo*, *kan* and *ery* stand for spectinomycin, chloramphenicol, neomycin, kanamycin and erythromycin resistance markers, respectively.

b CTSCCV, centre technique de la salaison, de la charcuterie et des conserves de viandes; ISHA, Institut Scientifique d'Hygiène et d'Analyse; BSFA strains were constructed during the “*Bacillus subtilis* functional analysis programme” [56].

### The formation of surface-associated submerged biofilms

The formation of surface-associated biofilms was performed in microtiter plates, as previously described with slight modifications [32]. Briefly, 250 µl of an overnight culture in TSB adjusted to an OD_600nm_ of 0.02 were added to the wells of the polystyrene 96-well microtiter plate with a μclear® base (Greiner Bio-one, France) which enables high resolution fluorescence imaging. The microtiter plate was kept at 30°C for 1∶30 to enable bacteria attachment to the bottom of the wells. After this adhesion step, the wells were rinsed with TSB to eliminate non-adherent bacteria and then refilled with 250 µl sterile TSB. The microtiter plate was then incubated for 48h at 30°C to allow biofilm development before structural analysis under the confocal laser scanning microscope (CLSM). Initial adhesion was also observed by transferring the microtiter plate under the CLSM directly after the 1∶30 adhesion step. In the case of GFP-carrying strains, isopropyl β-D-thiogalactopyranoside (IPTG) was added to the medium at a final concentration of 200µM to induce GFP expression.

### Macrocolony formation

To analyze colony architecture, 5 µl of an overnight culture in TSB were inoculated on 1.5% Tryptone Soya Agar (TSA, BioMérieux, France). When appropriate, the medium was supplemented with 200µM IPTG to allow GFP expression. The plates were then incubated at 30°C for 72h. Digital images of the colonies on the plates were taken using an Olympus C-5060 digital camera. The 3D architecture of macrocolonies of auto-fluorescent mutants was also observed by CLSM, as described in the section on *Confocal Laser Scanning Microscopy*.

### Pellicle experiments

After an overnight culture in TSB at 30°C, 10 µl of the bacterial suspension were used to inoculate 2 ml of TSB in 24-well plates (TPP, Switzerland). Each plate was then incubated at 30°C, and pellicle formation was recorded at 48h. Digital images of the pellicles in the wells of the plates were taken using an Olympus C-5060 digital camera.

### Swarming and swimming experiments

For swarming, 9 cm agar plates containing 20 ml TSB fortified with 0.8% agar were prepared and dried for 30 min with their lids open under a laminar flow hood. 10 µl of an overnight culture were then inoculated at the center of the swarming plates, dried for 15 min under the laminar flow hood and incubated at 30°C for 24h. For swimming, 9 cm agar plates containing 20 ml TSB fortified with 0.25% agar were prepared and dried for 30 min with their lids open under a laminar flow hood. 5 µl of an overnight culture were then inoculated at the center of the plate, dried for 15 min and incubated at 30°C for 24h. Digital images of the swarming and swimming plates were collected using an Olympus C-5060 digital camera. Each experiment was repeated three times.

### Confocal Laser Scanning Microscopy

Adhered cells, 48h-immersed biofilms and macrocolonies were observed using a Leica SP2 AOBS inverted confocal laser scanning microscope (CLSM, LEICA Microsystems, France) at the MIMA2 microscopy platform (http://voxel.jouy.inra.fr/mima2). For observations of adhered cells and immersed biofilms, the medium in the wells of the microplate was gently replaced with fresh TSB medium after the adhesion step or biofilm development to eliminate any free floating bacteria. Strains not carrying GFP constructions were tagged fluorescently in green with Syto9 (1∶1000 dilution in TSB from a Syto9 stock solution at 5mM in DMSO; Invitrogen, France), a nucleic acid marker. After 20 min of incubation in the dark at 30°C to enable fluorescent labeling of the bacteria, the plate was then mounted on the motorized stage of the confocal microscope. The microtiter plates were scanned using a 63×/1.2 N.A. water immersion objective lens. Fluorescent reporters excitation was performed at 488 nm with an argon laser, and the emitted fluorescence was recorded within the range 500–600 nm in order to collect Syto9 or GFP fluorescence (maximum emission of fluorescence at respectively 498 and 507 nm). To quantify the number of attached cells on the bottom of microplate wells, a total of nine images were acquired for each strain in three different wells. To generate images of the biofilms, a minimum of six Z- image series with a 1 µm step were acquired for each strain in three different wells. Furthermore, some images were acquired using a 10×/0.3 N.A. dry objective which enables a broader field of observations. Macrocolonies of GFP-carrying mutant strains were also observed using CLSM by transferring the colony from the agar plate to a glass slide which was then mounted upside-down on the stage of an inverted CLSM. The centers of macrocolonies were scanned using a 10×/0.3 N.A. dry objective and some Z-series images with a 1 µm step horizontal plane were also acquired.

### Analysis of CLSM images

Easy 3D blend projections of immersed biofilms or the structure of macrocolonies were reconstructed from Z-series images using IMARIS v7.0 software (Bitplane, Switzerland). The biovolumes of immersed biofilms were calculated using PHLIP [33], a freely available Matlab-based image analysis toolbox (http://phlip.sourceforge.net/phlip-ml). The biovolume represented the overall volume of a biofilm (in µm^3^) and could be used to estimate its total biomass. It was defined as the number of foreground pixels in an image stack multiplied by the voxel volume, which is the product of the squared pixel size and the scanning step size [34].

### Statistical analysis

One-way ANOVA were performed using Statgraphics v6.0 software (Manugistics, Rockville, USA). Significance was defined as a *P* value associated with a Fisher test value lower than 0.05.

## Results

### Different B. subtilis strains all form submerged surface-associated biofilms with structural heterogeneity

**Figure 1:**
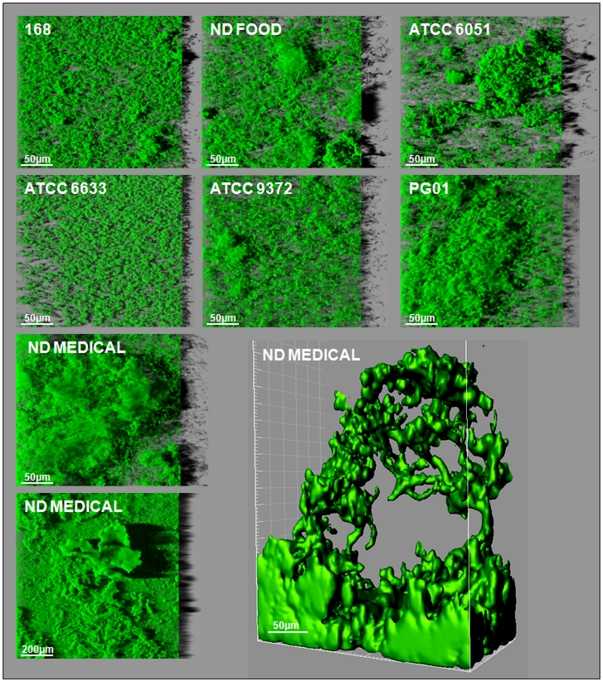
Three-dimensional biofilm structures obtained with the seven *B.subtilis* strains. These images present a representative, aerial, 3D view of the 48h-biofilm structures obtained with the seven *B. subtilis* strains using a microplate system, obtained from confocal image series using IMARIS software (including the shadow projection on the right). One iso-surface representation of a particular “beanstalk-like” structure is also shown for *B. subtilis* ND_medical_.

**Figure 2:**
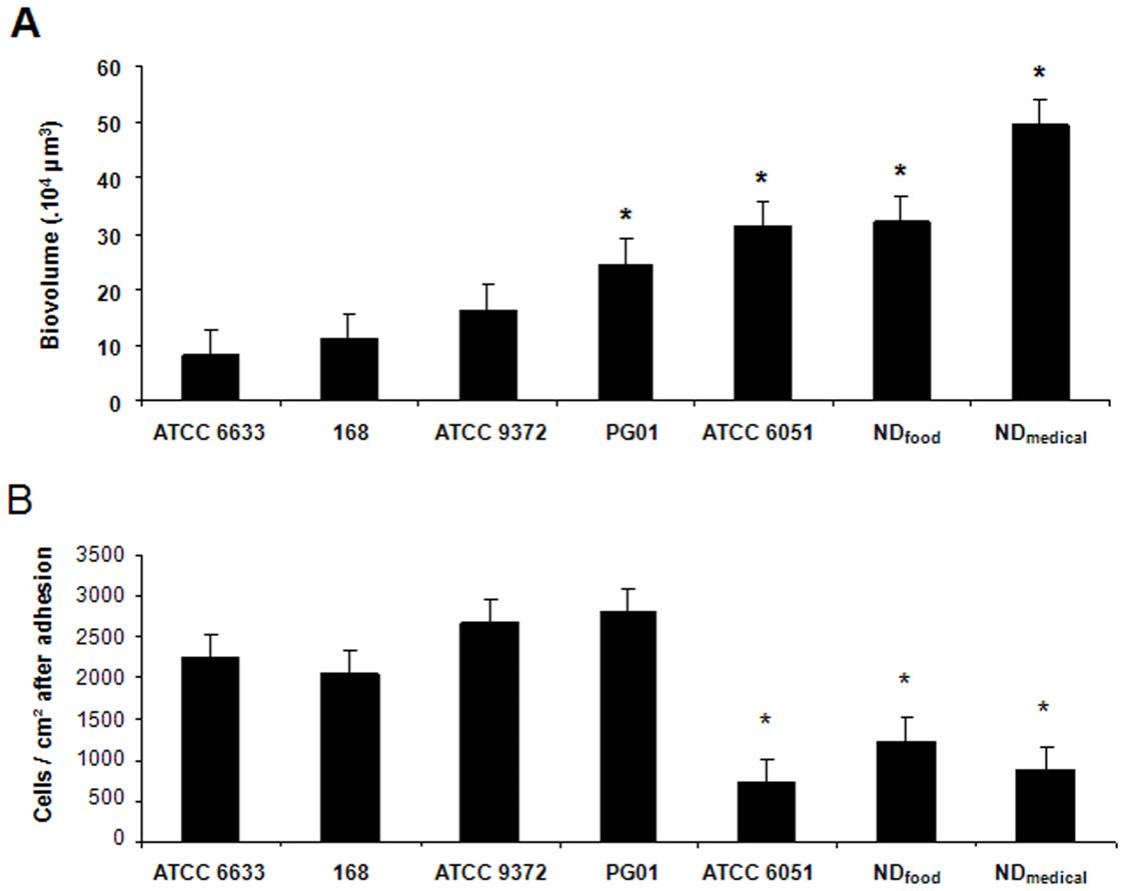
Biofilm biovolumes and the initial adhesion levels of the seven *B.subtilis* strains. (A) The biovolumes (µm^3^) in ascending order of the 48h-biofilms obtained with the seven *B. subtilis* strains in microtiter plates from confocal image series using the PHLIP tool. (B) Number of cells adhering per cm^2^ after 1h30 of adhesion in the microtiter plate. The error bars indicate the standard error and the statistically significant difference observed with strain 168 (*P*<0.05) is indicated by a star (*).

To characterize the ability of *B. subtilis* to adhere and form multicellular communities on surfaces, the three-dimensional structure of 48h biofilms formed by seven strains of different origins on the bottom surface of wells in a microtiter plate compatible with high resolution fluorescence imaging was analyzed using CLSM. Preliminary experiments demonstrated that biofilms reached their maximum biovolume within 24h to 48h, depending on strains considered (data not shown). Representative biofilm structures observed for the seven strains are presented in Fig. 1. The images correspond to three-dimensional blend reconstructions obtained from confocal images series with the dedicated IMARIS software, including virtual shadow projections on the right-hand side to represent biofilm sections. The associated biovolumes calculated from the series of images are presented in Fig. 2A. The results first of all showed the ability of all *B. subtilis* strains to form biofilms on the bottom of the microtiter plate wells. They also revealed a marked structural heterogeneity in the biofilms produced that depended on the strain considered. The non-domesticated strains, ND_food_ and ND_medical_, displayed the strongest biofilm-forming capacities under these growth conditions, and particularly the ND_medical_ strain which demonstrated a biovolume that was statistically significantly higher than that of all the other strains (*P*<0.05) (Fig. 2A). The biofilms obtained with this strain displayed dramatic, protuberant “beanstalk-like” structures that could reach a height of more than 300 µm, as shown at the bottom of Fig. 1. These structures appeared to consist of intertwined bundles of cells rising from the mat of cells on the bottom of the well and then congregating. While the other strains also produced clusters of various sizes, the structures they formed were much smaller than those displayed by ND_medical_. Interestingly, the three strains producing the biofilms with the highest biovolumes (ATCC 6051, ND_food_ and ND_medical_) showed a significantly smaller number of cells adhering to the bottom of the wells after 1h30 of adhesion when compared to the other strains (Fig. 2B).

**Figure 3:**
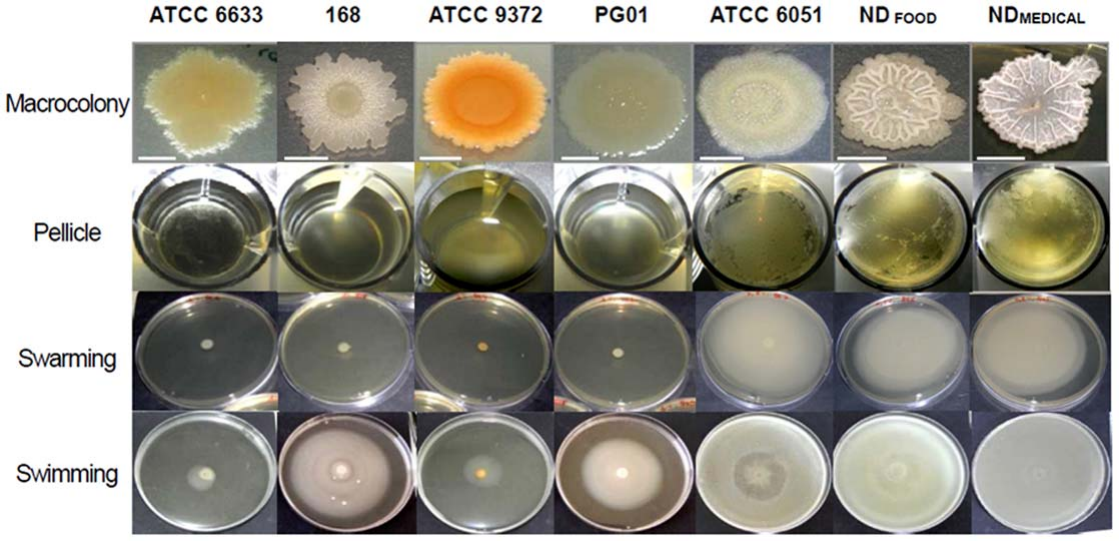
Macrocolony, pellicle, swarming and swimming phenotypes of the seven *B.subtilis* strains. Macrocolonies grown on TSA medium (1.5% agar) for 72h at 30°C after a central spot of 5 µl of an overnight bacterial culture in TSB. The scale bar is 5mm. Pellicles formed in 24-well microplates after 48h incubation at 30°C in TSB. Swarming plates (9 cm diameter) after 24h incubation at 30°C on TSA semi-solid medium (0.8% agar). Swimming plates (9 cm diameter) after 24h incubation at 30°C on TSA semi-solid medium (0.25% agar).

The ability of the seven strains to form macrocolonies and pellicles, to swarm and to swim was also evaluated and the results are presented in Fig. 3. These showed that only undomesticated strains, ND_food_ and ND_medical_, along with strain ATCC 6051, displayed a clear ability to swarm on agar, while the other four strains did not swarm at all. Interestingly, these three strains displayed the best swimming abilities and were the only strains to produce a pellicle at the air-liquid interface in the microtiter plate after 48h of incubation. The two undomesticated strains also formed markedly wrinkled colonies on the solid medium when compared to the other strains.

### Immersed biofilms and other B. subtilis multicellular models are under similar but not identical genetic controls

**Figure 4:**
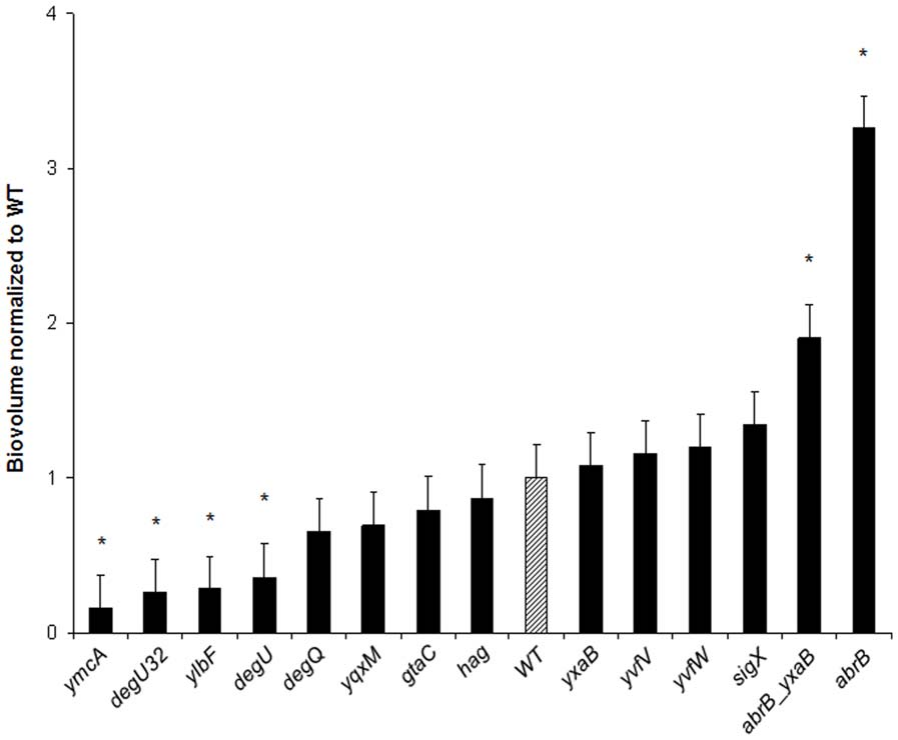
Biofilm biovolumes of the 14 *B.subtilis* mutant strains. Effects of mutations on the biofilm biovolumes of the 48h-biofilms obtained with the 14 mutants and wild-type strain (GM2812) (GFP-carrying strains). Biovolumes were normalized to wild-type (GM2812) and classified in ascending order. The error bars indicate the standard error and the statistically significant difference in biovolume obtained with the wild-type (*P*<0.05) is indicated by a star (*).

**Figure 5:**
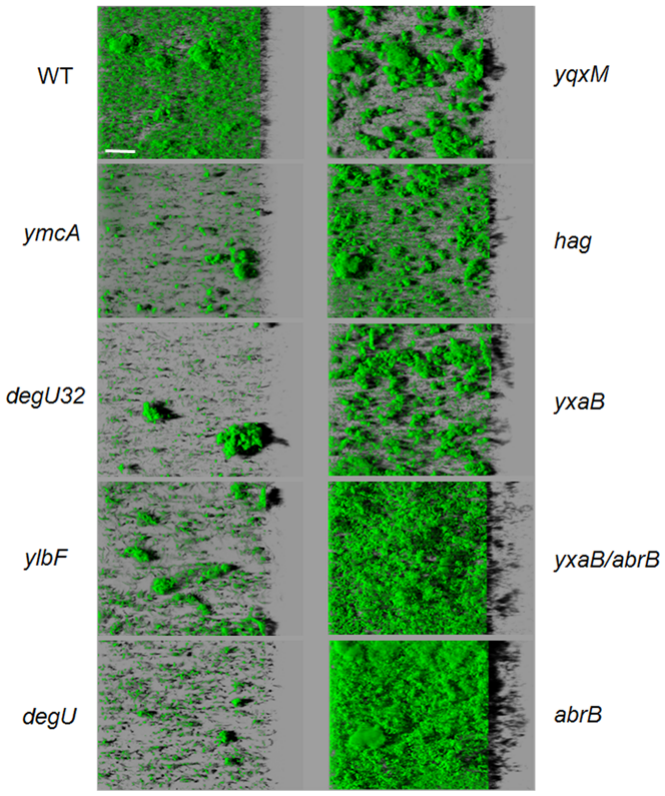
Effect of mutations on the three-dimensional structure of biofilms. Effects of mutations on immersed biofilm structures of the biofilms obtained with different GFP-carrying mutant strains and the corresponding reference wild-type (WT) strain from the confocal image series using the IMARIS software. Images depict an aerial view of 48h-biofilms in the microplate system. The scale bar is 50µm.

Our initial results demonstrated the ability of *B. subtilis* to form immersed surface-associated communities with biovolumes and macro-structures that differed as a function of the strain considered. We then investigated whether mutations known to affect biofilm formation in macrocolony or pellicle models would also affect it in our immersed surface-associated model. Thus volume and structure of the biofilms formed by different GFP-labeled mutants derived from strain 168 (see Table 1) were analyzed using CLSM (Fig. 4 and 5). We first checked whether GFP expression affected the biofilm forming ability of strain 168 by comparing the biovolume and structure of the 48h-biofilms of both wild-type and GFP-labeled strains. No significant differences were found between the biovolume values (data not shown). Moreover, as shown in Fig. 1 and Fig. 5, GFP expression did not appear to affect the three-dimensional structure of the biofilm.

The biovolumes of 48h-biofilms of *B. subtilis* mutants normalized to a wild-type biovolume are presented in Fig. 4. The results showed that six of the 14 mutations tested significantly affected the biofilm biovolume in the microtiter plate. Null mutations in the *ymcA* or *ylbF*, and *degU* KO or *degU32* alleles provoked a significant reduction in the immersed biofilm biovolume (*P*<0.05). The corresponding mutants did not form immersed biofilms in TSB in microtiter plates, despite the presence of few adhered cells and small aggregates, thus showing that these regulators are required for the formation of surface-associated *B. subtilis* communities under these conditions. By contrast, we observed that an *abrB* KO mutation strongly enhanced biofilm formation in our model. It should be noted that the *abrB* and *yxaB/abrB* mutants displayed 3.3-fold and 1.9-fold increases in biofilm biovolume, respectively, when compared to the wild-type strain (*P*<0.05) (Fig. 4). Although the biofilm biovolumes of *hag, yqxM* and *yxaB* KO mutants did not differ significantly from those of the wild-type, these mutations led to three-dimensional structural alterations of the immersed biofilms, as shown in Fig. 5. Deletion of the *hag* gene that codes for flagellin, a major component of the flagellum, did not impede the formation of a surface-associated biofilm under our conditions, but led to a more heterogeneous structure with a larger number of cellular aggregates. We found that the *yqxM* and *yxaB* mutants formed relatively independent clusters and did not cover the entire substratum available, unlike the wild-type strain. Moreover, in terms of initial adhesion, we found that only the *yqxM* mutation provoked a significant decrease in the number of adhered cells (31% reduction, *P*<0.05) compared to the wild-type (data not shown).

**Figure 6:**
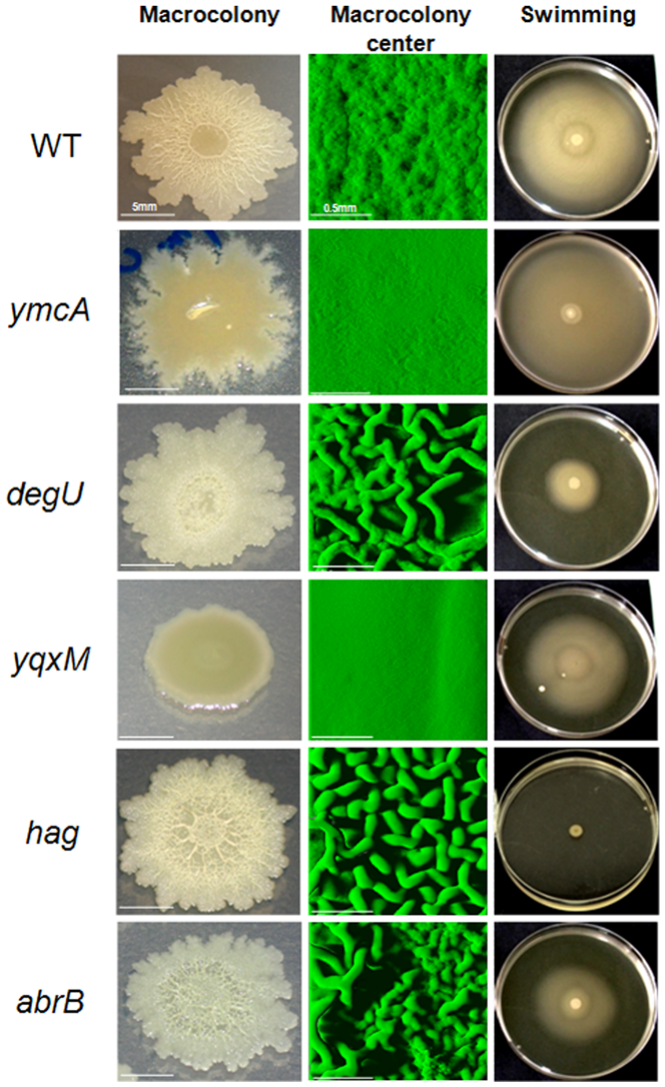
Effects of *ymcA*, *ylbF*, *degU*, *yqxM*, *hag* and *abrB* mutations on macrocolony and swimming phenotypes. Macrocolonies grown on TSA medium (1.5% agar) for 72h at 30°C. The left-hand column depicts macrocolony morphologies. The scale bar is 5mm. The middle column depicts aerial views of the centers of macrocolonies from confocal image series using IMARIS software. The scale bar is 0.5mm. The right-hand column shows the swimming plate (9 cm diameter, TSB+0.25% agar) after 24h of incubation at 30°C.

By comparison with the wild-type strain, both *ymcA* and *ylbF* mutants produced similar flat and mucoid colonies on agar, as shown for the *ymcA* mutant in Fig. 6. *degU* KO, and to a lesser extent *degU32*, induced the formation of wrinkles in the center of the colony and altered the swimming ability of the strains (Fig. 6 and data not shown). We found that both single *abrB* and double *yxaB/abrB* (not shown) mutants produced macrocolonies with a rough morphology and small wrinkles in the center, as well as altering swimming ability when compared to the wild-type strain, as shown in Fig. 6 for the *abrB* mutant. Interestingly, the *hag* mutation, which completely inhibited motility, also provoked the formation of wrinkles in the center of colonies, as observed for the *degU* mutants. While we did not find a marked impact of the *yxaB* mutation on macrocolony morphology when compared to the wild-type strain (data not shown), the *yqxM* mutation led to a flat and featureless colony with no horizontal outgrowths on the agar (Fig. 6).

## Discussion

Although the genetic pathways and mechanisms governing the formation of *B. subtilis* biofilms in air-liquid interface pellicle or colony models have been the subject of intensive study, little is known about biofilm development on solid surfaces. Indeed, since Hamon and Lazazzera [17] demonstrated the ability of *B subtilis* to form structures on abiotic surfaces, only a few studies have focused on immersed surface-associated models in order to study *B. subtilis* biofilm development [17], [18], [28], [30].

For this reason, we first studied the submerged biofilm architecture of seven *B. subtilis* strains of various origins using a microplate model. We found that not only were all the strains considered able to form a biofilm on the polystyrene bottom of microplate wells, but also that some of these strains developed “beanstalk-like” rising structures with an exceptional surface to volume ratio. This was particularly striking for the non-domesticated strain ND_medical_ that developed remarkable structures reaching more than 300µm in height. Indeed, we had previously showed that under similar growth conditions, *Pseudomonas aeruginosa* or *Staphylococcus aureus* strains, despite being known as excellent biofilm producers, did not form a biofilm more than 60 µm thick [32]. The ND_medical_ strain was isolated from endoscope washer-disinfectors because of its high resistance to oxidizing biocide treatment [35]. The authors suggested that compared to sensitive strains, this resistance is due to the high level of matrix production, as visualized by scanning electronic microscopy. It is therefore possible that the development of tall structures in our microtiter plate model using this strain was also related in part to a greater production of exopolymeric matrix. Interestingly, the three strains producing immersed biofilms with the highest biovolumes (ATCC 6051, ND_food_ and ND_medical_) were the only strains which displayed an ability to swarm and form an air-liquid pellicle after 48h of incubation. These observations were consistent with the existence of core mechanisms determinant for the development of multicellular communities, whatever the experimental model used. In addition, we found that these three strains exhibited the best swimming abilities. Previous studies on other species such as *Bacillus cereus*, *Escherichia coli*, *Listeria monocytogenes* or *Pseudomonas aeruginosa* [36], [37], [38], [39] had shown that motility could contribute to biofilm development through the recruitment of cells from the planktonic phase or through architectural structuring of the biological edifice. After 1h30 of adhesion, we found that the ATCC 6051, ND_food_ and ND_medical_ strains displayed a significantly smaller number of cells adhering to the bottom of the plate when compared with other strains displaying weaker swimming abilities. One explanation could be that, under our static conditions, bacteria could reach the bottom of the well by sedimentation so that the most motile cells were not necessarily advantaged in adhesion, as previously shown by Houry *et al.* [37] with *B. cereus*. These authors showed that the deletion of flagellum led to an increase in the bacteria bound to a glass surface in a flow-cell, and suggested that bacteria devoid of flagellum could have access to the substratum by sedimentation and then adhere better because the flagellum did not hinder contacts between the bacterial cell wall and the glass surface. Moreover, high motility could prevent the cells from staying for a sufficient time close to the substrate to initiate irreversible adhesion.

The experimental findings of our study suggested that development of the dramatic protruding structures appeared to be intimately related to the growth conditions applied here. Indeed, the ND_medical_ biofilms that formed in flow-cells under dynamic conditions were flat, compact and did not display “beanstalk-like” rising structures (data not shown), probably because of the shear forces induced by the flow. Moreover, other static systems such as immersed coupons did not seem to cause the development of such structures (data not shown). One hypothesis is that under our conditions, the small volume of TSB in the microtiter plate ensured a higher surface to volume ratio, and thus sufficient oxygenation of the medium for growth of the *B. subtilis* immersed biofilm. We also observed that repeated drastic rinsing, such as that performed during the widely employed crystal violet biofilm assay, led to a dislocation of these rising structures (data not shown). These complementary observations showed that our microtiter plate system and experimental conditions i.e. soft rinsing, might be appropriate to study the surface-associated community architecture of *B. subtilis* by providing beneficial conditions for the formation and preservation of immersed biofilms. This model could therefore be appropriate for the study of *B. subtilis* biofilms alongside pellicle and colony models, because it reflects other environmental conditions that may be encountered in natural, industrial or medical settings. Furthermore, this type of model associated with CLSM provides an opportunity to decipher the dynamic structure and reactivity of multicellular communities in a non-invasive manner, enabling a clearer understanding of structure/function relationships.

In order to better understand the mechanisms governing biofilm formation on immersed surfaces, different mutations known to affect biofilm formation in pellicle and macrocolony models were then introduced into a 168 derivative strain harboring a GFP-expression cassette. On the one hand, we found that *ymcA*, *ylbF* and *degU* KO and *degU32* alleles impeded biofilm formation in microtiter plate, indicating that these genes are required for *B. subtilis* biofilm formation and development on immersed surfaces. YmcA and YlbF are reported as regulators that are required for the formation of a macrocolony with complex surface features, or the formation of a pellicle at the air-liquid interface, because of their action on SinR activity that causes the derepression of matrix biosynthesis operons [19], [40]. It is therefore not surprising to observe that these regulators also affect the immersed surface-associated biofilm model because of their involvement in the central pathway for biofilm formation. The DegU transcription factor also coordinates the multicellular behavior of *B. subtilis* through the regulation of motility, poly-γ-glutamic acid [41] and protease production *via* a gradient in its phosphorylation level [22], [27], [28], [29]. The *degU32* mutation leads to a stabilization of the phosphorylated form of DegU [42]. In this context, a high level of DegU-P promotes the production of alkaline protease, levansucrase and other degradative exoenzymes, and inhibits motility and the production of flagella [43]. Thus the overproduction of proteases and other enzymes in the *degU32* mutant could impede matrix formation and structuring that is required for biofilm development, as shown by our observations in the microtiter plates. Conversely, previous studies had shown that an absence of the phosphorylated form of DegU led to non-activation of the genes required for mature colony or pellicle development [11], [22], [29]. Therefore, the gradual activation of DegU throughout its phosphorylation appears to be essential to the regulation of genes involved in the formation of immersed multicellular communities, as has been shown in other biofilm models.

On the other hand, we found that an *abrB* deletion enhanced biofilm biovolume when compared to the wild-type strain. This observation was consistent with the fact that AbrB was described as acting as a repressor of biofilm formation in previous studies [17], [18]. he suppression of AbrB could therefore provoke the over-expression of biofilm genes and lead to the increase in biofilm biovolume observed here. However, these authors found that a single *abrB* mutant produced a biofilm identical to that of the wild-type, even though they used a surface-associated immersed biofilm model. This difference could be explained by the different biofilm growth conditions and experimental protocols used during both studies. Indeed, Kobayashi [11] previously reported that an *abrB* mutant formed an air-liquid pellicle with an excessive architecture by comparison with the wild-type strain but only under certain medium conditions, thus underlining the dependence of the phenotype on the growth conditions used. We observed that the double *yxaB/abrB* mutant also displayed a higher biovolume than the wild-type, although this was smaller than that seen with the *abrB* single mutant (*P*<0.05). The *yxaB* single mutant produced a biofilm with a biovolume similar to that of the wild-type but with an altered three-dimensional structure. These results are consistent with the fact that *yxaB* is negatively regulated by AbrB and with the exopolysaccharide synthase function it may encode [44]. Indeed, the specific structure of the *yxaB* mutant biofilm may have been due to decreased exopolysaccharide synthesis.

Although the *yqxM* mutant formed a flat macrocolony without complex architecture, we did not find any quantitative difference between the immersed biofilm formed by this mutant and that of the wild-type, as previously observed by Hamon *et al.* [18]. Nevertheless, the *yqxM* mutant formed independent clusters and did not produce a mat of cells that entirely covered the surface, as observed for the wild-type. Furthermore, *yqxM* was the only mutant displaying a significantly smaller number of adhered cells after 1h30 of contact with the surface when compared to the wild-type strain. YqxM is the product of the first gene of the *yqxM-sipW-tasA* operon and appears to be involved in the proper localization of TasA to the matrix and its anchoring to the cell wall [13], [45], [46]. Romero *et al.* [15] recently reported that TasA forms amyloid fibers that are involved in the formation of a robust pellicle. Previous studies had shown that amyloid fibers (e.g. curli) can contribute to the attachment of bacteria to a surface or host cells and then participate in the three-dimensional structure of biofilms [47], [48], [49]. It is therefore possible that the specific structure of the *yqxM* mutant surface-associated biofilm may result from a weaker adhesion of the cells to the bottom of the well because of abnormal or absent TasA presentation to the cell envelope.

Interestingly, while flagella are required for the normal progression of pellicle formation [11], the flagellum deletion in the *hag* mutant did not lead to a reduction in initial cell adhesion and did not impact the biovolume of the immersed biofilm when compared to the wild-type strain. Nevertheless, the loss of flagella led to the formation of a more heterogeneous biofilm on the surface, with more clusters than the wild-type, suggesting that it plays a role in the architecture of communities. These results highlight the role of flagellum in the architectural development of submerged biofilms but not in initial adhesion to the substrate. The morphology of the center of the macrocolony suggested that motility also plays a role in macrocolony architecture. Indeed, while the wild-type strain produced a macrocolony with flat center, we observed that *hag* and *degU* null mutants, and with less pronounced phenotypes, *degU32*, single *abrB* and double *yxaB/abrB* mutants, produced a 72h-macrocolony with wrinkles in the center. Kobayashi [11] showed that a *degU* null mutant had few flagella and an *abrB* mutant containing a smaller number of flagella than that observed for the wild-type. Similarly, Amati *et al.* [43] showed that expression of the *hag* gene was also markedly reduced in a *degU32* mutant. Furthermore, our results confirmed that all these mutations caused changes to swimming abilities when compared to the wild-type strain. Interestingly, Watnick *et al.* [50] made similar observations for *Vibrio cholerae*, showing that flagellar mutants displayed rugose colonies. The authors presented evidence to suggest that the absence of flagellum results in increased EPS production in colonies, leading to the rough morphologies observed. Kobayashi [11] also suggested that flagellum proteins may play a regulatory role rather than a direct role (such as on adhesion or cell-cell interaction) in pellicle formation by *B. subtilis*.

To conclude, the surface-associated submerged model proposed here produced a diversity of *B. subtilis* biofilm architectures and the existence of remarkable protruding “’beanstalk-like” structures with some strains. As in pellicle or macrocolony models, YmcA, YlbF, DegU and AbrB regulators play a key role in the development of these immersed structures, probably because of their implication in central biofilm regulation pathways such as matrix biosynthesis. Conversely, we found that some mutations known to affect macrocolony organization or pellicle development did not markedly impede biofilm formation on a surface. The possible coexistence of an immersed biofilm on the bottom of a plate and a pellicle at the air-liquid interface in the same well raises the question of the spatial and temporal relationships between the two communities, a point that will be investigated in future studies.
